# Investigation of factors influencing sugar consumption in early life: a cross-sectional study

**DOI:** 10.1590/1807-3107bor-2025.vol39.062

**Published:** 2025-06-02

**Authors:** Tainá Fontes de SOUZA, Aline Gama FREITAS, Mariana Leonel MARTINS, Andréa FONSECA-GONÇALVES

**Affiliations:** (a) Universidade Federal do Rio de Janeiro – UFRJ, School of Dentistry, Department of Pediatric Dentistry and Orthodontics, Rio de Janeiro, RJ, Brazil.; (b) Universidade Federal do Rio de Janeiro – UFRJ, School of Dentistry, Rio de Janeiro, RJ, Brazil.

**Keywords:** Sugars, Diet, Breast Feeding, Child, Dental Caries

## Abstract

This study evaluated the influence of socioeconomic factors, caregiver characteristics, and early feeding practices on sugar consumption frequency (FSC) during early childhood. Data were collected from dental records of children aged 1 to 5 years old, who were attended at CliBin^®^, including sex, age, skin color, income level, mother’s age and years of education, primary caregiver, prior instructions on caries prevention, type of early feeding practices (breastfeeding/formula/mixed), exclusive breastfeeding (EB) up to 6 months, children’s dental care and their consumption of sugar-sweetened beverages (SSB) and cookies/sugar (CS), with frequency classified as: never (2 points), ≤ 3 times/day (1 point) and > 3 times/day (0 points). The lower the median score the higher the FSC. Descriptive analysis, Kruskal-Wallis test, and ANOVA were applied considering p < 0.05. A total of 123 records were included. The children were predominantly male (56.1%), and brown (46.5%), with an average age of 2.1 (± 0.9) years. The mother was the primary caregiver (86.1%), with ≤ 30 years of age (52.0%), < 12 years of education (75.2%), and previously instructed on caries prevention (58.2%). Most children were breastfed (45.5%) for 23.2 (±9.7) months on average, received EB (87.1%), consumed SSB (87.6%) and CS (90.9%) 3 times/day. The total FSC mean score was 1.6 ± 0.9 (95%CI: 1.4–1.7). Children of younger mothers (≤ 30 years) had the lowest FSC scores (1.4 ± 0.9; 95%CI: 1.0–1.6; p=0.01). It was observed that among the factors studied, only mothers in the younger age range influenced high sugar consumption during early childhood.

## Introduction

Sugar consumption among children is a global concern due to its direct relationship with the development of non-communicable chronic diseases, such as dental caries, obesity, and cardiovascular diseases, throughout an individual’s life.^1-3^ A review of national diet surveys across the world showed that the percentage of daily energy intake from total sugars is highest in infants under 4 years old, highlighting the importance of controlling sugar consumption among children. Thus, an effort has been made to curb sugar consumption globally, by imposing additional taxes on products containing sugar such as the sugar-sweetened beverages (SSB). However, the Brazilian government has deviated from this measure by not implementing it, and reducing taxes on SSB, leaving the population more vulnerable to the health impacts of sugar.^5^

High-sugar diets are a crucial factor in the development of dental caries, as frequent exposure to sugar causes an imbalance within the dental biofilm, making it more aciduric and acidogenic, which leads to the production of acids that, when in contact with dental surfaces, may result in caries lesions.^6^ Therefore, understanding sugar consumption patterns is essential for developing measures to prevent unhealthy dietary habits that lead to diseases, including dental caries.

The preference for sugar and the aversion to bitter food are seen as innate human characteristics, reflecting a biological drive towards foods that are calorie- and protein-dense to produce energy and protection against toxic foods.^7^ Additionally, the human sugar-oriented eating behavior begins to form in the womb, when the fetus detects tastes and flavors from the mother’s diet through the amniotic fluid, around the last trimester of pregnancy, due to the maturity of the olfactory and gustatory system.^8^ The high-sugar diet continues to develop throughout early childhood, when the initial forms of feeding to which the child is exposed usually are breastfeeding, formula feeding, or mixed feeding. Thus, the type of feeding also shapes the child’s diet, with breastfeeding being an extremely powerful means to promote a healthy life^9^ and associated with healthier eating habits in childhood.^10^

External factors also play a fundamental role in dietary patterns during early childhood. The social environment in which the child grows significantly affects the development of their eating habits.^11^ Culture, lifestyle, education, and parents’ economic status can directly influence children’s eating habits^12,13^ and how parents offer sugary foods to them. Therefore, this study aimed to investigate the influence of socioeconomic factors, caregiver characteristics, and early feeding practices on the frequency of sugar consumption in early childhood.

## Methods

### Study design and settings

This is a cross-sectional study, part of a project previously approved by the Research Ethics Committee of the Clementino Fraga Filho University Hospital (protocol number 4.916.767). This study was designed based on STROBE criteria,^14^ and aimed to evaluate data from dental records of infants and preschool children treated at a public school clinic of a public university (CliBin^®^, Faculty of Dentistry of the Federal University of Rio de Janeiro, Brazil).

### Sample size and participants

The sample consisted of all dental records of active patients from CliBin^®^, recorded from June 2015 to January 2023. Dental records of patients aged 1 to 5 years who had no motor and systemic impairments were included. Records that did not include information regarding variables of interest, such as eating habits and sugar consumption, were excluded.

### Variables and data source

The following data were collected from anamnesis and clinical examination records:

#### a.Dependent variables:

Sugar consumption: The consumption frequency of sugar-sweetened beverages (SSB) and the consumption frequency of cookies and sugar were classified according to the frequency most associated with the development of caries, as found by Feldens et al.,^15^ and was scored as follows: never (2 points), consumption up to 3 times/day (1 point), and consumption more than 3 times/day (0 points). A total sugar consumption score or frequency of sugar consumption (FSC) was achieved by adding the SSB and cookies/sugar consumption scores (0–4 points). The lower the median score, the higher the children’s frequency of sugar consumption. Scores from 0 to 2 were considered high consumption and between 2.1 and 4 as low consumption.

#### b.Independent variables:

Socioeconomic factors: sex (female/ male); age (≤ 2 years old, > 2 years old); skin color (white, brown, black, or other); income level based on Brazil’s economic classification criteria^16^ divided into upper (A), middle (B1, B2) and lower (C1, C2, D-E)^17^; siblings (yes/no).

Caregiver characteristics: primary caregiver (parents or other); mother’s age (≤30 years or > 30 years); mother’s years of education (≤ 12 years or > 12 years); caregiver previously received instructions on how to prevent dental caries (yes/ no).

Feeding practices: type of feeding practice received until the time of examination (breastfeeding, formula feeding, or mixed feeding); duration of the feeding practices (duration in months of breastfeeding, formula feeding or mixed feeding; exclusive breastfeeding until six months of age (yes/no).

#### c.Covariates:

Sugar consumption: consumption of sugar-sweetened beverages (SSB) (yes/no); consumption of cookies and sugar (yes/no).

Dental care characteristics: first visit to the dentist (yes/no); frequency of tooth brushing (< 3×/day, 3 ×/day, or more); use of toothpaste (without fluoride/ with less than 1000 ppm fluoride/with 1,000 to 1,500 ppm fluoride)^18^, who performs the brushing (caregiver/ child/ child and caregiver); brushes teeth before bedtime (yes/no); eats or drinks between the last brushing and bedtime (yes/no); family history of dental caries (yes/ no); experience of dental caries calculated by the mean number of decayed, missing and filled deciduous teeth (dmft)^19^; and the presence of dental cavities considering the “d” component > 0 (zero) (yes/no) of the dmft index.

The dental records were completed by postgraduate pediatric dentistry students and checked by trained and calibrated professors (Cohen’s Kappa coefficient = 0.91 ± 0.37 for inter-examiner agreement regarding the dmft caries index).

## Statistical methods

All data collected were tabulated and analyzed using IBM SPSS Statistics software version 24.0 (SPSS Inc., Chicago, USA). Descriptive analyses were performed using means and absolute frequencies. Non-normality of data was confirmed by the Kolmogorov-Smirnov test (p = 0.00). Thus, the Kruskal-Wallis test was used to evaluate the influence of independent data on the frequency of sugar consumption scores. The statistical significance level for all tests was set as p ≤ 0.05.

## Results

A total of 454 dental records were evaluated, of which 331 were excluded for not meeting the eligibility criteria. Therefore, 123 records were included in this study.

### Socioeconomic characteristics

The socioeconomic characteristics of the sample are presented in Table 1. The average age of the children was 2.1 (± 0.9) years, with the majority being ≤ 2 years old (65.9%). They were predominantly male (56.1%), and their caregivers declared them as brown (46.5%). Most of them belonged to the middle socioeconomic class (60.0%) and had, on average, 1.0 (± 1.2) siblings (57.7%).

**Table 1:** Socioeconomic characteristics, feeding practices, sugar consumption habits and dental characteristics of the sample.

Variables	n	%
Socioeconomic characteristics		
Sex (n = 123)		
Female	54	43.9
Male	69	56.1
Child’s age (n = 123)		
≤ 2 years	81	65.9
> 2 years	42	34.1
Skin color (n = 114)		
White	47	41.2
Brown	53	46.5
Black	13	11.4
Others	1	0.9
Economic class (n = 120)		
Upper	2	1.7
Middle	72	60.0
Lower	46	38.3
Siblings (n = 119)		
Yes	71	59.7
No	48	40.3
Caregiver characteristics		
Primary caregiver (n = 122)		
Mother	105	86.6
Father	6	4.9
Other	11	9.0
Mother’s age (n =123)		
≤ 30 years	64	52.0
> 30 years	59	48.0
Mother’s years of education (n = 113)		
≤ 12 years	85	75.2
> 12 years	28	24.8
Received instructions on how to prevent dental caries (n = 122)		
Yes	71	58.2
No	51	41.8
Feeding practices		
Type of feeding (n = 122)		
Breastfeeding	56	45.5
Formula feeding	33	27.0
Mixed feeding	33	27.0
Exclusive breastfeeding until six months (n = 70)		
Yes	61	87.1
No	9	12.9
Sugar consumption habits		
Consumes SSB* (n = 121)		
Yes	106	87.6
No	15	12.4
Consumption frequency of SSB* (n = 114)		
Never	16	14.0
≤ 3 times/day	56	49.1
> 3 times/day	42	36.8
Eats cookies and sugar (n = 121)		
Yes	110	90.9
No	11	9.1
Consumption frequency of cookies and sugar (n=115)		
Never	11	9.6
Up to 3 times/day	77	67.0
More than 3 times/day	27	23.5
Dental care characteristics		
First visit to the dentist (n = 122)		
Yes	74	61.2
No	47	38.8
Family history of dental caries (n = 123)		
Yes	99	80.5
No	24	19.5
Use of fluoride toothpaste (n = 92)		
Without fluoride	12	13.0
= 1,000 to 1,500 ppm F	78	84.8
< 1,.000 ppm	2	2.2
Frequency of toothbrushing (n = 121)		
< 3 times/day	71	58.7
≥ 3 times/day	50	41.3
Who performs the brushing (n = 121)		
Caregiver	93	76.9
Child	4	3.3
Child and caregiver	24	19.8
Use of dental floss (n =1 22)		
Yes	18	14.8
No	104	85.2
Consumes food or drinks between last brushing and bedtime (n = 117)		
Yes	74	63.2
No	43	36.8
Presence of dental caries lesions (n = 123)		
Yes	61	50.4
No	60	49.6

### Caregiver characteristics

Their primary caregivers were their parents (91.0%), with mothers being the most frequent (n = 105/86.1%). The average age of the mothers was 30.3 (± 6.7), who had 12 years of education or less (75.2%) and had previously received instructions on how to prevent dental caries (58.2%).

### Type of feeding

As shown in Table 1, most children were exclusively breastfed until 6 months of age (87.1%). They were more breastfed (45.5%) than fed with formula (26.8%) or mixed feeding (26.8%) (Table 1). The average duration of breastfeeding was 23.2 (±9.7) months, while formula feeding had an average of 25.5 (±8.8) months, and mixed feeding was the longest (27.5±7.8 months).

### Sugar consumption habits

Most children consumed SSB (87.6%) up to 3 times/day (49.1%), achieving an average score of 1.8 ± 0.4 (95%CI: 1.7–1.9) for consumption. Additionally, they also consumed cookies and sugar (90.9%) up to 3 times/day (67.0%), with an average score of 1.9 ± 0.6 (95%CI: 1.7–2.0). The mean total score for the consumption of SSB, cookies, and sugar (FSC) was 1.6 ± 0.9 (95%CI: 1.4–1.7).

### Dental care characteristics

Most children were on their first visit to the dentist (61.2%), had their teeth brushed by their caregiver (76.9%) less than 3 times/day (58.7%), and used toothpaste with the correct fluoride concentration (1,000 to 1,500 ppm) (84.8%). Most did not use dental floss (85.2%) and usually ate or drank between the last brushing and bedtime (63.2%). Most of their family members had experienced dental caries (80.5%), but half of the children (49.6%) did not have any dental caries lesions. The average dmft score among children was 3.1 (± 4.3).

### Influence of variables on the frequency of sugar consumption score

Among all the socioeconomic, caregiver, and type of feeding characteristics assessed, only the mother’s age influenced the sugar consumption score (p=0.01), with younger mothers (≤ 30 years) (52.0%) appearing to offer sugar more frequently to their child (1.4 ± 0.9; 95%CI: 1.1–1.6), as shown in Table 2. The presence of dental caries lesion was associated (p = 0.03) with higher FSC, with an average of 1.4 ± 0.9 (95%CI:1.1–1.6) points.

**Table 2:** Frequency of children’s sugar consumption (mean score) according to the variables studied.

Variables	Mean score (SD)	95% CI	p-value
Socioeconomic characteristics			
Sex			
Female	1.5 (0.9)	1.2-1.8	0.56
Male	1.7 (0.9)	1.4-1.9	
Child’s age			
≤ 2 years old	1.6(0.9)	1.4-1.8	0.74
> 2 years old	1.5(1.0)	1.2-1.9	
Skin color			
White	1.8 (0.9)	1.5-2.0	0.11
Brown	1.4 (0.8)	1.1-1.6	
Black	1.9 (1.0)	1.2-2.6	
Other	2.0 (0.0)	NA	
Economic class			
Upper	0.5 (0.7)	-5.8-6.8	0.98
Middle	1.5 (1.0)	1.2-1.7	
Lower	1.8 (0.8)	1.5-2.0	
Siblings			
Yes	1.6 (0.9)	1.3-1.7	0.41
No	1.7 (1.0)	1.3-2.0	
Caregiver characteristics			
Primary caregiver			
Mother	1.2 (0.5)	1.4-1.8	0.10
Father	1.2 (0.5)	0.0-1.6	
Other	2.0 (1.4)	0.2-2.8	
Mother’s age			
≤ 30 years old	1.4 (0.9)	1.0-1.6	0.01*
> 30 years old	1.8 (0.9)	1.5-2.1	
Mother’s years of education			
≤ 12 years	1.6 (0.9)	1.3-1.7	0.87
> 12 years	1.7 (1.1)	1.2-2.1	
Received instruction on how to prevent dental caries			
Yes	1.6 (0.9)	1.3-1.8	0.78
No	1.6 (1.6)	1.3-1.9	
Feeding practices			
Type of Feeding			
Breastfeeding	1.7 (1.0)	1.3-1.9	0.60
Formula feeding	1.7 (0.9)	1.3-2.0	
Mixed feeding	1.5 (0.9)	1.1-1.8	
Exclusive breastfeeding until six months			
Yes	1.7 (0.9)	1.4-1.9	0.60
No	1.4 (1.5)	-0.4-3.2	
Dental cavities			
Presence			
Yes	1.4(0.9)	1.1-1.6	0.03*
No	1.8(0.9)	1.5-2.0	

* Statistically significant p-value.

## Discussion

This study investigated the influence of socioeconomic factors, caregiver characteristics, and feeding practices on sugar consumption during early childhood, as these variables have the potential to shape and develop pathways that lead to the adoption of a cariogenic diet.

Among all the variables evaluated, the mother’s age stood out in influencing children’s sugar consumption, with mothers under 30 years old offering sugary foods more frequently than older mothers. This finding is consistent with studies conducted by Toloni et al.^20^, Reis et al.^21^, Tovar et al.,^22^ and Feldens et al.^23^ In Tovar et al.^22^‘s study, mothers who graduated from high school or who had further education had an income of ~ $20,000 (dollars)/year. Despite differences in educational level and socioeconomic characteristics observed by Tovar et al.^22^ compared to the present study and other studies cited, all identified that the consumption of sugary beverages and foods at an early age was higher in children of younger mothers. Furthermore, a systematic review based on the analysis of cross-sectional studies from developed countries indicated that the higher consumption of sugary drinks in children up to 6 years old was associated with younger parental age.^12^

Maternal age can significantly impact how mothers offer sugary foods to their children. Younger mothers are more likely to be inexperienced with motherhood and tend to be more insecure when dealing with the child’s demands.^23^ Therefore, they have greater difficulty recognizing the child’s hunger and satiety^24^ cues, consequently leading to more frequent offerings of ultra-processed foods ^21^ and sugary foods^23^ to their children, as they tend to soothe the child.^23^ Additionally, older mothers who are over 30 years of age, are more likely to have experienced dental caries during their childhood, as the prevalence of this disease was higher in the past ^24,25^. Therefore, they are more cautious about their child’s sugar consumption, attempting to prevent the disease from affecting the child too. However further studies are needed to explore the reasons why sugar consumption is more frequent amongst children of younger mothers.^22^

Furthermore, as shown in this study, mothers are usually the primary caregivers for children and are nowadays widely integrated into the job market, which leaves them less time to dedicate themselves to domestic activities compared to the past. This may influence the way they feed their children,^26^ possibly resulting in increased consumption of processed foods, which are quicker to prepare but contain more sugar.

Most children evaluated in this study had at least one sibling. Although the number of children did not stand out as an influential factor in sugar consumption within the sample, Reis et al.^21^ demonstrated that children living with more people at home tend to consume more sugar.

Certain conditions and characteristics are unconditionally related to the mother’s age and directly influence the child’s sugar consumption. One of these interconnected factors is that young mothers may have a lower level of knowledge compared to older ones, which was observed in this study regarding caregiver characteristics, with the majority consisting of mothers under 30 years old and with less than 12 years of education. This set of characteristics can directly reverberate in the way they offer sugary foods to their children, as they may be fully unaware of the importance of establishing a sugar-controlled diet from an early age, especially during the first thousand days of life and early childhood, to prevent the development of non-communicable chronic diseases that derive from a high-sugar diet, such as dental caries.^27^ Although most of the caregivers evaluated in this research reported having received some instruction on preventing caries, it is possible that the source of the information received was not a properly trained professional, as the majority of the children evaluated were on their first visit to the dentist.

In addition to the years of education of family members, socioeconomic class is a determinant factor for a child’s healthy diet,^12^ as eating habits are directly related to the family’s financial status. The child’s socioeconomic status will indicate what the environment in which they grow up will be like; what quantity and quality of food the child will have access to and will be exposed to.^28,29^ Most children in this research belonged to the middle class, and there is a global trend of higher sugar consumption in populations from middle- and low-income countries.^30^ Moreover, a survey conducted by Sigh et al.^28^ in 187 countries showed that the consumption of SSB was higher in middle-income countries compared to either high-income or low-income countries, highlighting the need for greater attention to sugar consumption in this economic group.

Since the diet can be influenced by several variables in a dynamic process^21^, the impact of early feeding practices on the frequency of sugar consumption was also evaluated in this study. No significant relationship was found between the type and duration of feeding and the frequency of sugar consumption. However, a recent systematic review with infants demonstrated a possible association between breastfeeding and healthier eating habits later in life.^6^ Additionally, this review demonstrated a negative association between the consumption of foods high in added sugars and eating habits^9^. Nonetheless, due to the heterogeneity of the studies evaluated, solid evidence for such observations has not been found yet, which demonstrates the need for studies in this area.

The present results must be reproduced with caution, considering their limitations. The information was collected from a very restricted population composed mainly of middle-class mothers over 30 who received preventive guidance and had the opportunity to exclusively breastfeed their babies during the first 6 months. Besides, they refer to a specific region of the country, which may not represent the majority of the Brazilian population, where social vulnerability is high and, consequently, access to information and guidance on dental care is limited.^31^ In addition to that, as this study was designed cross-sectionally, we identified the associations between the investigated factors and the frequency of sugar consumption, rather than the causal inference. The small sample and the limitation of incomplete data obtained from the dental records may also have directly influenced the results. Therefore, studies with larger populations are required to produce more reliable results.

Another point to mention is that the present study did not investigate when and with what device the children began cleaning their teeth. This issue could have influenced the experience of caries in the sample but may not have interfered with the children’s eating habits. Thus, future studies addressing this issue should be conducted.

The findings of this study emphasize the significant relationship between the frequency of sugar consumption and the presence of dental caries lesions. Restricting the frequency of sugar consumption is part of the necessary care to prevent the occurrence of dental caries diseases and must be combined with proper oral hygiene, ideally performed after main meals using toothpaste with a fluoride concentration of 1,000 to 1,5000 ppm.^18^ In the present study, although most children used toothpaste with the ideal fluoride concentration, they consumed food between the last brushing and bedtime. Besides, despite the WHO recommendation to avoid sugar consumption until the age of 2 to prevent dental caries ^1,27^, the studied population had an average age of 2.1 (± 0.9) years and a high frequency of sugar consumption was observed, which is the opposite of the recommendation.

The dmft index found in this study was greater than that found in 5-year-old children examined in the last national survey carried out in Brazil.^31,32^ This result emphasizes the need to invest in and improve programs such as the National Oral Health Policy (Brasil Sorridente), which can disseminate preventive information to the population. Furthermore, additional taxation on products containing sugar should be implemented. This study also showed that younger mothers can influence their children’s sugar consumption, emphasizing the need to closely monitor this group during dental care, and highlighting the importance of a sugar-free diet before the age of two. Thus, understanding the mechanisms that lead to frequent sugar consumption should be elucidated with larger studies to develop prevention programs that can help avoid the factors leading to this pattern.

## Conclusion

Although the results found should not be extended to the entire Brazilian population, it was observed that younger mothers offered sugar to their children more often than older ones. Therefore, among the socioeconomic factors, caregiver characteristics, and early feeding practices, only the mother’s age influenced sugar consumption in early childhood.
